# Randomized, double-blind, placebo-controlled study of the efficacy of four probiotics to modify the risk for postoperative complications in colorectal surgery

**DOI:** 10.1186/cc14470

**Published:** 2015-03-16

**Authors:** K Kotzampassi, G Stavrou, G Damoraki, M Georgitsi, G Basdanis, G Tsaousi, EJ Giamarellos-Bourboulis

**Affiliations:** 1 Aristotle University of Thessaloniki, Greece; 2 University of Athens Medical School, Athens, Greece

## Introduction

Heterogeneous published Results led us to conduct a clinical trial to assess the efficacy of a new formulation of four probiotics (P) as prophylaxis for complications after colorectal surgery.

## Methods

A double-blind, placebo-controlled randomized study was conducted enrolling patients undergoing colorectal cancer surgery. Placebo or a formulation of *L. acidophilus*, *L. plantarum*, *B. lactis *and *S. boulardii *was administered starting 1 day before operation and continuing for 15 days post operation. Patients were followed-up for 30 days with the development of postoperative complications as the primary outcome. PAXGene tubes and serum were collected on postoperative day 4 for measurement of gene expression and serum cytokines (http://ClinicalTrials.gov NCT02313519).

## Results

**Figure 1:**
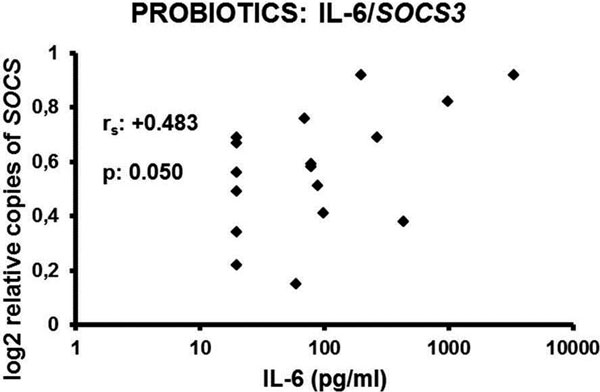


Administration of P significantly decreased the rate of all postoperative major complications (28.6% vs. 48.8% of placebo, *P *= 0.010, odds ratio: 0.42). Major benefit was found in the reduction of the postoperative pneumonia rate (2.4% vs. 11.3%, *P *= 0.029), of wound infections (7.1% vs. 20.0%, *P *= 0.020), of anastomotic leakage (1.2% vs. 8.8%, *P *= 0.031) and of the need for mechanical ventilation (20.2% vs. 35.0%, *P *= 0.037). The time until hospital discharge was shortened as well. Gene expression of SOCS3 was positively related with circulating IL-6 in the P group but not in the placebo group (Figure 1).

## Conclusion

The studied P formulation significantly decreased the risk of postoperative complications, namely mechanical ventilation, infections and anastomotic leakage. Modulation of the gene expression of SOCS3 is one suggested mechanism.

